# Single-photon emission computed tomography (SPECT) predicted neurological prognosis in heat stroke: A case report

**DOI:** 10.1016/j.heliyon.2023.e18285

**Published:** 2023-07-20

**Authors:** Keisuke Suzuki, Kazuyuki Miyamoto, Takahiro Kanai, Mariko Kurihara, Kazuki Kikuchi, Kohei Harano, Akihito Kato, Masaharu Yagi, Yoshimitsu Ohgiya, Kenji Dohi

**Affiliations:** a Department of Emergency and Disaster Medicine, Showa University School of Medicine, 1-5-8 Hatanodai, Shinagawa-ku, Tokyo 142-8666, Japan; b Department of Radiology, Division of Radiology, Showa University School of Medicine, Shinagawa-ku, Tokyo 142-8666, Japan; c Department of Emergency and Disaster Medicine, Showa University, Yokohama Northern Hospital, 35-1 Chigasaki Chuo Tsuzuki-ku, Yokohama 224-8503, Japan

**Keywords:** Heat stroke, MRI, SPECT, Cerebellar ataxia, Case report

## Abstract

Heat stroke may cause multi-organ dysfunction and death. Some patients with neurological abnormalities in the acute phase have neurological sequelae, particularly cerebellar ataxia, in the recovery phase. However, there is no method to predict the neurological prognosis, and the usefulness of imaging has not yet been established. We report the case of an 86-year-old woman with dementia brought to our emergency department in a coma and hyperthermia. The patient was diagnosed with heat stroke and promptly treated in the ICU but remained unconscious. The patient gained consciousness on day 19, but difficulty with stillness associated with cerebellar ataxia in her right upper extremity became apparent. On day 1, head magnetic resonance imaging (MRI) showed no obvious abnormality. However, on day 6, high-signal areas, suggestive of edema, were seen in the bilateral cerebellar hemispheres. Single-photon emission computed tomography (SPECT) on day 9 revealed significant hypoperfusion in the right cerebellum. These changes improved at the time of hospital discharge. This was a case of persistent cerebellar ataxia due to heat stroke, in which imaging findings improved over time. In most cases, MRI findings do not match clinical symptoms. However, the low cerebral blood flow in the early SPECT images was consistent with the clinical symptoms. MRI may not be a prognostic indicator; however, SPECT images may be useful for predicting sequelae.

## 1 Introduction

Heat stroke is a systemic disease caused by high temperature exposure; its incidence is estimated to increase due to climate change [1]. Heat stroke may cause multi-organ dysfunction or death, and the central nervous system disorder rate is particularly high [2,3]. Further, 23% of patients with neurological abnormalities in the acute phase have neurological sequelae in the recovery phase, 71% of whom have long-term cerebellar ataxia [4].

However, no methods to predict the neurological prognosis exist, and imaging usefulness has not yet been established. We report a case of heat stroke in which neurological prognosis was predicted using single-photon emission computed tomography (SPECT).

## 2 Case report

**Fig. 1:**
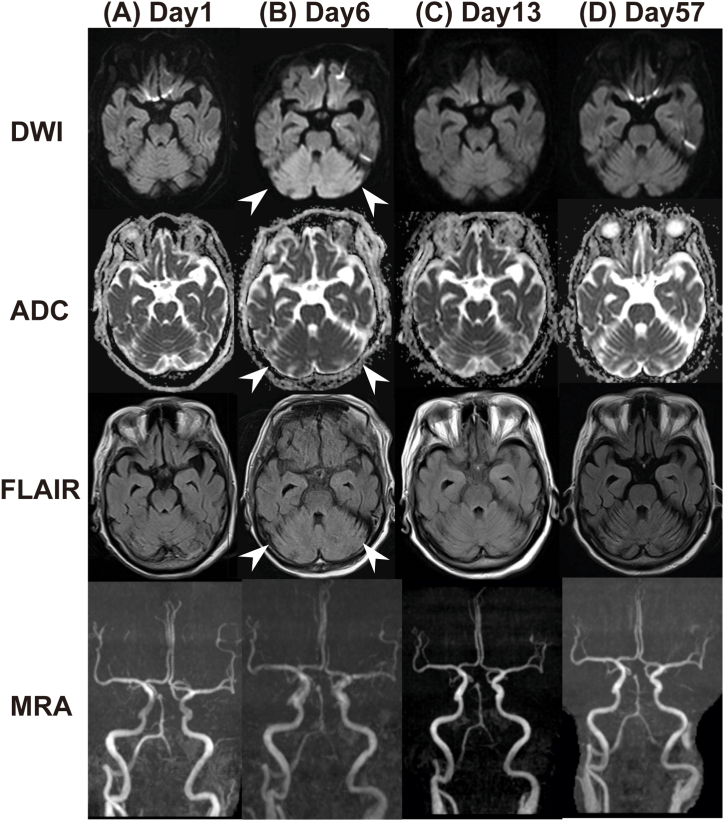
Chronological series of DWI, ADC, and FLAIR images of the patient's brain. (A) The initial MRI showed no obvious abnormal signal intensity. (B) On day 6, a high-intensity signal was observed on DWI and FLAIR (arrowhead), with a low-intensity signal on ADC in the bilateral cerebellar hemispheres (arrowhead). (C) On day 13, the high-intensity signals on DWI were reduced. (D) On day 57, the high-intensity signals on DWI had disappeared, and those on FLAIR had reduced. MRI, magnetic resonance imaging; FLAIR, fluid-attenuated inversion recovery; DWI, diffusion-weighted imaging.

An 86-year-old woman with dementia (no internal medication) lived in a non-air-conditioned room during scorching summer. She was found in an unconscious state by her family and transferred to the emergency department (ED). Upon arrival, she was in a coma, with a Glasgow Coma Scale score (GCS), 6 (E1V1M4); body temperature, 42.4 °C; blood pressure, 88/40 mmHg; and pulse rate, 120 beats/min. The patient had hepatic dysfunction, renal dysfunction, and disseminated intravascular coagulation syndrome (DIC) (Table 1). Head magnetic resonance imaging (MRI) and magnetic resonance angiography (MRA) showed no obvious abnormality (Fig. 1A).

**Table 1:** Laboratory data on arrival. WBC, white blood cell; Hb, hemoglobin; Plt, platelet; BUN, blood urea nitrogen; Cre, creatinine; T-bil, total bilirubin; D-bil, direct bilirubin; AST, aspartate aminotransferase; ALT, alanine aminotransferase; CRP, C-reactive protein; PT-INR, prothrombin time-international normalized ratio; APTT, activated partial thromboplastin time; FDP, fibrin degradation products; TSH, thyroid stimulating hormone; FT3, free triiodothyronine; FT4, free thyroxine.

Laboratory Data	
WBC (/μL)	15,200
Hb (g/dL)	11.2
Plt (/μL)	75,000
BUN (mg/dL)	25.3
Cre (mg/dL)	1.92
T-bil/D-bil (mg/dL)	1.7/1.0
AST/ALT (U/L)	194/49
CRP (mg/dL)	0.08
PT-INR/APTT	1.60/34.7
FDP/D-dimer (μg/dL)	74.51/31.07
TSH (μU/mL)	76.01
FT3 (pg/mL)	1.04
FT4 (ng/dL)	<0.4
Vitamin B1 (ng/mL)	18.2

**Fig. 2:**
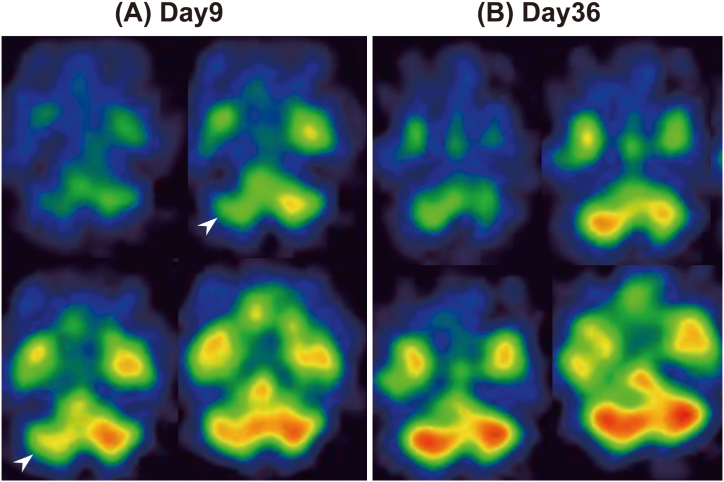
Single photon emission computed tomography using N-Isopropyl-p-[123I] iodoamphetamine (123I-IMP) perfusion scans on days 9 and 36. (A) On day 9, SPECT showed decreased CBF in the right cerebellar hemispheres (arrowhead). (B) On day 36, areas of hypoperfusion had disappeared.

She was diagnosed with severe heat stroke, with an Acute Physiology and Chronic Health Evaluation (APACHE) II score, 42 and sequential organ failure assessment (SOFA) score, 15. She was intubated and admitted to the ICU. Aggressive extracorporeal cooling, fluid resuscitation, and DIC treatment were initiated immediately. In addition, fursultiamine and levothyroxine were supplemented. However, she remained unconscious. On day 6, axial diffusion-weighted (DW) MRI showed high-signal areas, suggestive of edema, in the bilateral cerebellar hemispheres. On day 9 (Fig. 1B), SPECT using N-isopropyl-p-[^123^I] iodoamphetamine (^123^I-IMP) showed hypoperfusion in the right cerebellum (Fig. 2A). MRI findings improved on day 13 (Fig. 1C) and recovered to E4VTM6/GCS after day 19.

SPECT findings using ^123^I-IMP improved on day 36 (Fig. 2B). She continued her rehabilitation, and as her muscle strength improved, she was weaned from the ventilator. However, on day 49, difficulty with stillness associated with cerebellar ataxia in her right upper extremity became apparent for the first time. On day 57, MRI changes disappeared (Fig. 1D), and she was transferred to the hospital on day 88 without right cerebellar ataxia recovery.

Written consent was obtained from the patient's family and patient's personal information was anonymized with great care. The patient provided informed consent for the publication of her case history.

## 3 Discussion

We encountered a case of persistent right cerebellar ataxia due to heat stroke. In this case, SPECT findings were more consistent with clinical findings than MRI findings.

In this case, Wernicke's encephalopathy and Hashimoto's encephalopathy were also considered in the differential diagnosis but were not aggressively suspected based on imaging findings and clinical symptoms. The main MRI findings in Wernicke's encephalopathy were reported to be in the area around the third ventricle and the middle cerebral aqueduct, and in Hashimoto's encephalopathy, cerebral white matter lesions and limbic system lesions were reported to be the main findings [5,6].

Heat stroke damages Purkinje cells in the cerebellum, which have low tolerance to heat. In mouse heat stroke models, Purkinje cell numbers decreased from the first week after heat stroke and did not improve even after 9 weeks of observation [7]. Several reports have shown abnormal signals on MRI owing to heat stroke, many of which show high DWI signals in the cerebellar hemispheres, as in the present case [8,9]. However, in most cases, MRI findings do not match clinical symptoms.

The imaging findings herein were bilateral; however, the symptoms were unilateral. MRI images also improved; therefore, it was not a cerebellar infarction. There was no change in MRA over time, and spasm involvement was negative. Heat stroke increases blood-brain barrier (BBB) permeability not only by overproduction of cytokines but also by brain temperature [10]. The result was angioedema and possibly cellular edema. Heat stroke shares key pathophysiological events with reversible posterior occipital lobe leukoencephalopathy. It is postulated to cause diverse signal patterns due to vascular dysregulation, BBB disruption, and associated brain edema [9].

SPECT is excellent for evaluating brain hemodynamics. It is useful for diagnosing and differentiating dementia, including Alzheimer's disease, and is widely used in clinical practice. SPECT using ^123^I-IMP has high contrast resolution and is considered useful for evaluating blood flow distribution [11]. Cerebral blood flow (CBF) decreases by 30% after exposure to heat and when the deep-body temperature reaches 40 °C [12]. In this case, not only dehydration but also the heat stroke itself may have caused the decrease in CBF. Herein, CBF was significantly reduced in the right cerebellar hemisphere during the acute phase but improved over time. However, the reduced blood flow may have caused mild ischemia, leading to Purkinje cell death. Therefore, they may not have remained as infarct foci on MRI. Surprisingly, the low cerebellar blood flow in the early SPECT images was consistent with the clinical symptoms. MRI may not be a prognostic indicator; however, SPECT images may be useful for predicting later sequelae.

## 4 Conclusion

MRI and SPECT imaging changes observed during the acute phase of heat stroke improved during the chronic phase. MRI findings were nonspecific and did not correlate with clinical symptoms, whereas early SPECT findings were associated with clinical symptoms. Our findings show that SPECT imaging may be useful for predicting sequelae.

## CRediT authorship contribution statement

Keisuke Suzuki: Writing–original draft, conceptualization. Kazuyuki Miyamoto wrote the original draft of the manuscript. Takahiro Kanai: Writing–original draft and data curation. Mariko Kurihara: Review and editing. Kazuki Kikuchi: Review and editing. Kohei Harano: review and editing. Akihito Kato: Data curation. Masaharu Yagi: Writing, reviewing, and editing. Yoshimitsu Ohgiya: Supervision. Kenji Dohi: Supervision.

## Author contribution statement

All authors listed have significantly contributed to the investigation, development and writing of this article.

## Data availability statement

All relevant data pertaining to this case are contained in the article.

## Patient consent

The patient and family provided informed consent for the publication of her case history.

## Sources of support

This work was partially supported by JSPS KAKENHI [Grant Number 22K16646 (K. S.)].

## Declaration of competing interest

The authors declare that no potential conflict of interest that could have appeared to influence the work reported in this paper.
